# Individual and Combined Effects of Booting and Flowering High-Temperature Stress on Rice Biomass Accumulation

**DOI:** 10.3390/plants10051021

**Published:** 2021-05-20

**Authors:** Aqib Mahmood, Wei Wang, Iftikhar Ali, Fengxian Zhen, Raheel Osman, Bing Liu, Leilei Liu, Yan Zhu, Weixing Cao, Liang Tang

**Affiliations:** 1 National Engineering and Technology Center for Information Agriculture, Nanjing Agricultural University, Nanjing 210095, China; aqib2028@gmail.com (A.M.); 2018201094@njau.edu.cn (W.W.); ifi.agronomist@gmail.com (I.A.); 2017201081@njau.edu.cn (F.Z.); raheel_osman@yahoo.com (R.O.); bingliu@njau.edu.cn (B.L.); liuleilei@njau.edu.cn (L.L.); yanzhu@njau.edu.cn (Y.Z.); caow@njau.edu.cn (W.C.); 2 Key Laboratory for Crop System Analysis and Decision Making, Ministry of Agriculture and Rural Affairs, Nanjing Agricultural University, Nanjing 210095, China; 3 Engineering Research Center for Smart Agriculture, Ministry of Education, Nanjing Agricultural University, Nanjing 210095, China; 4 Jiangsu Key Laboratory for Information Agriculture, Nanjing Agricultural University, Nanjing 210095, China; 5 Jiangsu Collaborative Innovation Center for Modern Crop Production, Nanjing Agricultural University, Nanjing 210095, China

**Keywords:** booting, flowering, multiple high-temperature stress, photosynthetic production, biomass, yield, rice

## Abstract

Extreme temperature events as a consequence of global climate change result in a significant decline in rice production. A two-year phytotron experiment was conducted using three temperature levels and two heating durations to compare the effects of heat stress at booting, flowering, and combined (booting + flowering) stages on the production of photosynthates and yield formation. The results showed that high temperature had a significant negative effect on mean net assimilation rate (MNAR), harvest index (HI), and grain yield per plant (YPP), and a significant positive effect under treatment T_3_ on mean leaf area index (MLAI) and duration of photosynthesis (DOP), and no significant effect on biomass per plant at maturity (BPP_M_), except at the flowering stage. Negative linear relationships between heat degree days (HDD) and MNAR, HI, and YPP were observed. Conversely, HDD showed positive linear relationships with MLAI and DOP. In addition, BPP_M_ also showed a positive relationship with HDD, except at flowering, for both cultivars and Wuyunjing-24 at combined stages. The variation of YPP in both cultivars was mainly attributed to HI compared to BPP_M_. However, for biomass, from the first day of high-temperature treatment to maturity (BPP_T-M_), the main change was caused by MNAR followed by DOP and then MLAI. The projected alleviation effects of multiple heat stress at combined stages compared to single-stage heat stress would help to understand and evaluate rice yield formation and screening of heat-tolerant rice cultivars under current scenarios of high temperature during the rice-growing season.

## 1. Introduction

Rice (*Oryza sativa* L.) is the primary source of food for the majority of the world’s population and is grown under a wide range of environmental conditions [1]. Global warming not only enhances the mean temperature but also intensifies the frequency and severity of heat stress events in rice-growing regions [2,3], where the present temperature is already close to the critical threshold level for rice production [4,5]. Consequently, any further increase in temperature beyond the threshold levels, particularly during critical growth stages, will cause significant grain yield losses [1,6,7].

The processes of photosynthesis and yield formation are controlled by temperature, but a rise in air temperature that exceeds a critical level for a certain amount of time causes irreversible and adverse changes in plant production and growth [8,9]. However, the sensitivity and intensity of the impact of heat stress are generally dependent on the crop growth stages and physiological processes [10]. Recent studies revealed that short-term heat stress during the reproductive growth period has a negative impact on rice growth and grain yield formation [11,12,13,14], and this negative impact on yield reduction is higher than that on yield losses due to seasonal warming losses in global rice production regions [14]. Another significant determinant of the effect of heat stress is the duration of high-temperature exposure. The rice yield, for example, declines almost linearly as the time of high-temperature exposure increases. In addition, the critical duration to induce equivalent sterility is shortened at higher temperatures [11,12]. Due to the relationship between temperature intensity and duration, a cumulative temperature above a threshold value appears to have a greater effect [15,16].

According to the source-sink theory, yield formation is governed by photosynthetic efficiency and biomass accumulation (the two most sensitive crop growth processes affected by heat stress) [17,18]. Crop photosynthetic production is determined by leaf photosynthetic rate, leaf area expansion, and photosynthetic duration. Short-term heat stress during the reproductive period results in the assimilation of photosynthates in vegetative parts by limiting the sink capacity [19,20]. Under such circumstances, leaf senescence is generally delayed, which in turn increases the leaf area expansion, photosynthetic efficiency, and duration in the late grain-filling stages of rice plants [11]. It has been previously reported that major yield losses were mainly attributed to limited nutrient translocation rather than a reduction in photosynthetic production under high temperatures [12,21,22].

Functional growth analysis offers a useful framework to assess a crop’s capability to capture growth-restricted resources and the efficiency with which they can be used to produce biomass. Generally, these analyses involve using repeated observations to plot specific functions describing changes in primary crop growth parameters over time. In many cases, crop growth analysis uses leaf area as a morphological yield component and leaf photosynthetic efficiency as a physiological yield component [23,24]. Moreover, grain yield can be measured via functional growth analysis using photosynthetic property parameters, including leaf area index, net assimilation rate, and photosynthesis duration [23,25]. A significant decline in rice grain yield as a consequence of a reduction in the net assimilation rate under high temperature has been previously reported [26,27]. Therefore, the net assimilation rate is considered to be the major cause of yield losses under high-temperature stress because it is a highly important parameter representing photosynthetic potential and dry matter accumulation of the crop [21,28]. However, previous studies have focused largely on the impact of high temperatures on rice photosynthesis and yield [11,12,29,30], with less emphasis on rice biomass production and the relative contribution of biomass production to grain yield.

Plants respond and adapt to abiotic stress by triggering a series of reactions at molecular, cellular, and physiological levels [31,32]. Therefore, it is important for the production of heat-tolerant varieties to consider how rice plants adapt to heat stress. Attempts have been made to establish thermo-tolerance in the plant by pre-treatment using a high sub-lethal temperature prior to a lethal temperature [33,34,35]. However, few attempts have been made using pre-treatment with accompanying lethal followed by lethal temperatures in rice. In addition, earlier studies have primarily centered on the effect of high temperatures on rice at a single growth stage [11,12,36,37] rather than investigating the impact of multiple high-temperature stresses at two critical growth stages.

Therefore, this study endeavored to compare the impacts of heat stress during booting, flowering, and combined stages (booting + flowering) on the processes of biomass accumulation and grain yield using functional growth analysis. We aimed to investigate the relative contribution of photosynthetic property parameters to the variability of biomass production and grain yield under high-temperature conditions and to establish the quantitative relationships between photosynthetic property parameters, biomass accumulation, grain yield, and heat degree days (HDD), which combine the intensity and duration of heat stress. The projected results would help to understand and evaluate yield formation under global climate change and in the screening of heat-tolerant rice cultivars.

## 2. Results

### 2.1. Effect of High Temperature at Booting, Flowering, and Combined Stages on Photosynthetic Property Parameters

#### 2.1.1. Effect of High Temperature on Mean Leaf Area Index

**Figure 1:**
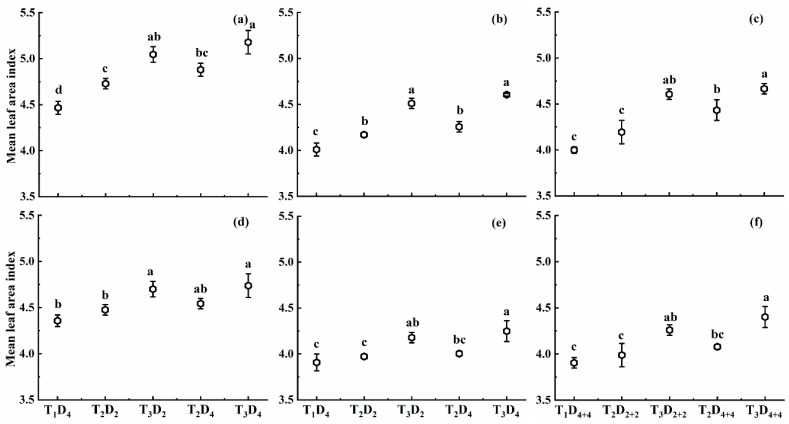
Mean leaf area index (MLAI) under different high-temperature treatments at booting, flowering, and combined stages during the 2016–2017 growing seasons. (**a**) Huaidao-5 at booting treatment; (**b**) Huaidao-5 at flowering treatment; (**c**) Huaidao-5 at combined stages treatment; (**d**) Wuyunjing-24 at booting treatment; (**e**) Wuyunjing-24 at flowering treatment; (**f**) Wuyunjing-24 at combined stages treatment. Different letters indicate significant differences at the 0.05 level. Vertical bars represent the standard deviation of the mean.

High-temperature treatment at booting, flowering, and combined stages increased the MLAI of both rice cultivars (Figure 1). The increase in MLAI was significant in combined-stages stress, followed by booting and flowering stages. The increase in MLAI for T_2_ and T_3_ compared to T_1_D_4_ (control) at booting, flowering, and combined stages for Huaidao-5 was 5.7–15.6%, 3.7–14.6%, and 5.8–17.7%, respectively. In contrast, Wuyunjing-24 showed a smaller increase in MLAI than Huaidao-5, with the increase in MLAI up to 2.7–8.7%, 1.6–8.7%, and 2.1–12.7% for treatments T_2_ and T_3_, compared to T_1_, for booting, flowering, and combined stages, respectively (Figure 1). Under high-temperature conditions, MLAI showed a greater increase at combined stages than booting and flowering stages. For an increase of 1 °C in high temperature at booting, flowering, and combined stages, MLAI increased by 1.3%, 1.2%, and 1.5% for Huaidao-5, and increased by 0.7%, 0.7%, and 1.0% for Wuyunjing-24, for 4 days’ duration for individual stages and 8 days’ duration for combined stages (Figure 1).

At the same high-temperature level, MLAI increased with increasing high-temperature duration for both cultivars at booting, flowering, and combined stages. MLAI increased by 1.4%, 1.2%, and 0.4% for Huaidao-5, and increased by 0.4%, 0.9%, and 0.9% for Wuyunjing-24 under T_3_, respectively, for each one-day increase in high-temperature duration at booting, flowering, and combined stages (Figure 1). Overall, the effect of high temperature on MLAI was significant for the high-temperature level at booting, flowering, and combined stages, whereas the effect of high-temperature duration and interaction of high-temperature level and duration was not significant (Table 1).

#### 2.1.2. Effect of High Temperature on Mean Net Assimilation Rate

**Figure 2:**
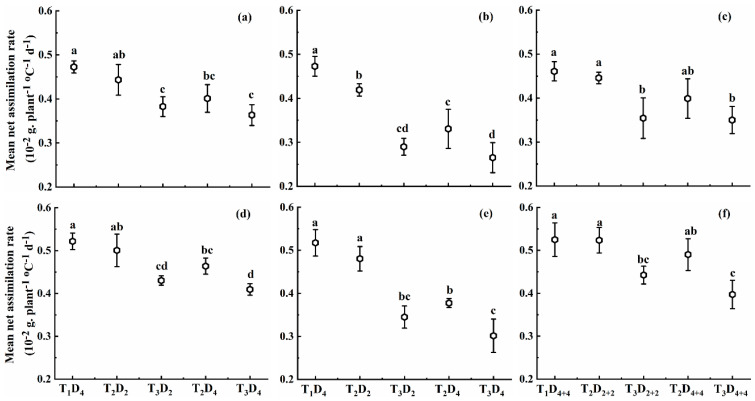
Mean net assimilation rate (MNAR) under different high-temperature treatments at booting, flowering, and combined stages during the 2016–2017 growing seasons. (**a**) Huaidao-5 at booting treatment; (**b**) Huaidao-5 at flowering treatment; (**c**) Huaidao-5 at combined stages treatment; (**d**) Wuyunjing-24 at booting treatment; (**e**) Wuyunjing-24 at flowering treatment; (**f**) Wuyunjing-24 at combined stages treatment. Different letters indicate significant differences at the 0.05 level. Vertical bars represent the standard deviation of the mean.

High temperatures at booting, flowering, and combined stages decreased MNAR in both cultivars (Figure 2). MNAR at booting, flowering, and combined stages under temperature levels T_2_ and T_3_ declined by 6.2–23.1%, 11.3–44.0%, and 3.3–24.0% for Huaidao-5, and 4.0–21.5%, 7.1–41.7%, and 0–24.0% for Wuyunjing-24, respectively, compared to T_1_D_4_ (control). These results reveal that MNAR was more sensitive to high temperature at flowering than combined and booting stages. In addition, MNAR decreased with increasing temperature levels for both cultivars. For an increase of 1 °C in high temperature at booting, flowering, and combined stages, the MNAR decreased by 1.9%, 3.7%, and 2.0% for Huaidao-5, and decreased by 1.7%, 3.5%, and 1.8% for Wuyunjing-24, for 4 days’ duration for individual stages and 8 days’ duration under combined stages (Figure 2). The adverse effect of increasing high-temperature levels on MNAR of Huaidao-5 was greater than that of Wuyunjing-24 at all stages.

When high temperature remained the same, MNAR decreased with increasing high-temperature duration at every stage and for both cultivars. MNAR declined by 4.5%, 9.4%, and 2.5% under T_2_ and 2.1%, 2.7%, and 0.2% under T_3_ for Huaidao-5, whereas for Wuyunjing-24, it decreased by 3.5%, 9.9%, and 1.6% under T_2_ and 2.0%, 4.2%, and 2.2% under T_3_, respectively, for each one-day increase in high-temperature duration at booting, flowering, and combined stages (Figure 2). Increasing temperature levels at all growth stages significantly decreased MNAR. However, the duration and interaction between temperature level and duration depicted significant variation only at the flowering stage for both cultivars (Table 1).

#### 2.1.3. Effect of High Temperature on Duration of Photosynthesis

**Figure 3:**
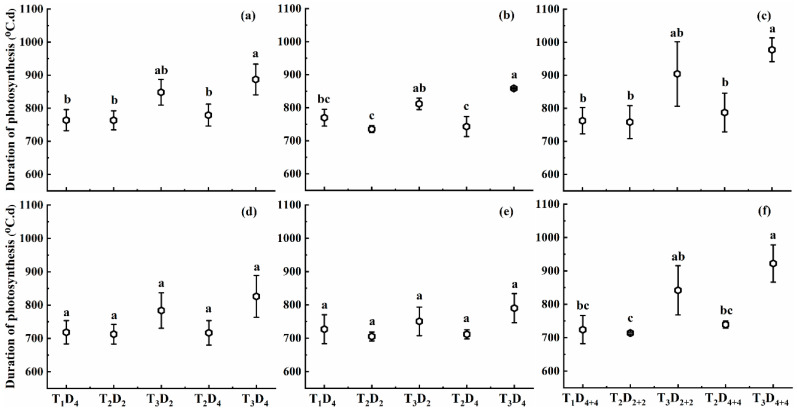
Duration of photosynthesis (DOP) under different high-temperature treatments at booting, flowering, and combined stages during the 2016–2017 growing seasons. (**a**) Huaidao-5 at booting treatment; (**b**) Huaidao-5 at flowering treatment; (**c**) Huaidao-5 at combined stages treatment; (**d**) Wuyunjing-24 at booting treatment; (**e**) Wuyunjing-24 at flowering treatment; (**f**) Wuyunjing-24 at combined stages treatment. Different letters indicate significant differences at the 0.05 level. Vertical bars represent the standard deviation of the mean.

The changes in the duration of photosynthesis under different high-temperature treatments are displayed in Figure 3. These results reveal that the DOP showed a decreasing trend with increasing temperature for T_2_; however, it exhibited an increasing trend with increasing duration for T_3_. The DOP at booting, flowering, and combined stages under temperature level T_3_ increased by 11.0–16.3%, 5.5–11.4%, and 18.6–28.6% for Huaidao-5, and 9.1–14.5%, 3.2–8.5%, and 16.3–27.4% for Wuyunjing-24, respectively, compared to T_1_D_4_ (control). Furthermore, DOP showed different patterns of findings under temperature level T_2_: it increased during the booting and combined stages for D_4_ and D_4+4_, respectively, but decreased during the flowering stage for both durations. The increase in DOP was obvious at combined stages followed by booting and flowering stages, and this increase was higher in Huaidao-5 compared to that of Wuyunjing-24. For an increase of 1 °C in high temperature at booting, flowering, and combined stages, the DOP increased by 0.9%, 0.5%, and 0.9%, and 1.4%, 0.9%, and 2.4% for Huaidao-5, and increased by 0.8%, 0.3%, and 1.4%, and 1.2%, 0.7%, and 2.2% for Wuyunjing-24 under 2 and 4 days’ duration for individual stages and 4 and 8 days duration under combined stages, respectively (Figure 3).

The effect of high-temperature duration on DOP differed between the two cultivars and treatment stages when the high-temperature level remained the same. DOP increased by 2.7%, 3.0%, and 2.5% for Huaidao-5 and increased by 2.7%, 2.7%, and 2.8% for Wuyunjing-24 under T_3_, for each one-day increase in high-temperature duration at booting, flowering, and combined stages, respectively (Figure 3). Additionally, the effect of high temperature on DOP was only significant for high-temperature levels in both cultivars at all growth stages, except for Wuyunjing-24 at flowering, where the DOP variation was non-significant. In contrast, the effect of high-temperature duration and interaction of high-temperature level and duration was not significant for both cultivars (Table 1).

#### 2.1.4. Effect of High Temperature on Harvest Index

**Figure 4:**
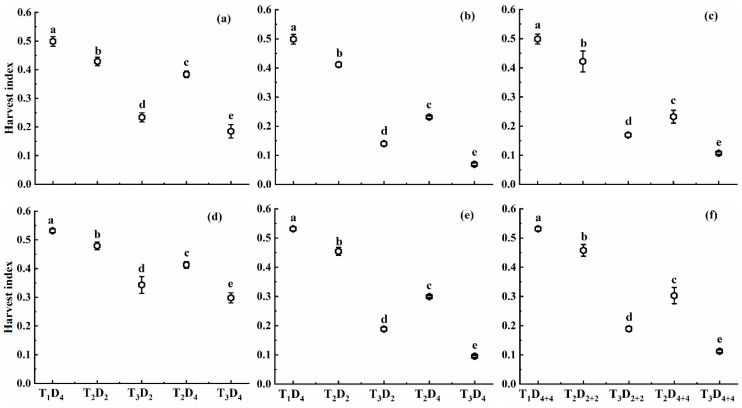
Harvest index (HI) under different high-temperature treatments at booting, flowering, and combined stages during the 2016–2017 growing seasons. (**a**) Huaidao-5 at booting treatment; (**b**) Huaidao-5 at flowering treatment; (**c**) Huaidao-5 at combined stages treatment; (**d**) Wuyunjing-24 at booting treatment; (**e**) Wuyunjing-24 at flowering treatment; (**f**) Wuyunjing-24 at combined stages treatment. Different letters indicate significant differences at the 0.05 level. Vertical bars represent the standard deviation of the mean.

High temperatures also resulted in a decreased harvest index in both cultivars and with a trend similar to the pattern of MNAR (Figure 4). Generally, HI decreased as the high-temperature level and duration increased. A lower HI was observed under high-temperature treatments at flowering followed by combined and booting stages. The decline in HI at booting, flowering, and combined stages under temperature levels T_2_ and T_3_ was 14.0–62.8%, 17.5–86.1%, and 15.4–78.6%, and 9.9–44.0%, 14.5–82.1%, and 13.9–78.9% for Huaidao-5 and Wuyunjing-24, respectively, compared to T_1_D_4_ (control). This pattern indicates that Huaidao-5 was more affected by high-temperature than Wuyunjing-24, and flowering was the most vulnerable stage. Moreover, HI decreased with increasing temperature levels for both cultivars. For an increase of 1 °C in high temperature at booting, flowering, and combined stages, the HI decreased by 4.1%, 5.5%, and 5.0%, and 4.9%, 7.1% and 6.5% for Huaidao-5, and decreased by 2.7%, 4.9%, and 4.8%, and 3.5%, 6.6%, and 6.4% for Wuyunjing-24 under 2 and 4 days’ duration for individual stages and 4 and 8 days’ duration for combined stages.

When high temperature remained the same, HI decreased with increasing high-temperature duration at every stage and for both cultivars. HI declined by 4.5%, 18.1%, and 9.5% under T_2_, and 4.8%, 7.1%, and 3.1% under T_3_ for Huaidao-5, whereas for Wuyunjing-24, it decreased by 6.3%, 14.6%, and 14.6% under T_2_, and 4.3%, 8.8%, and 7.2% under T_3_, respectively, for each one-day increase in high-temperature duration at booting, flowering, and combined stages (Figure 4). Increasing temperature level, duration, and their interaction showed a significant decrease in HI at all growth stages, except Huaidao-5 at booting, which depicted a non-significant variation for the interaction between high-temperature level and duration (Table 1).

#### 2.1.5. Effect of High Temperature on Biomass Accumulation and Grain Yield

**Figure 5:**
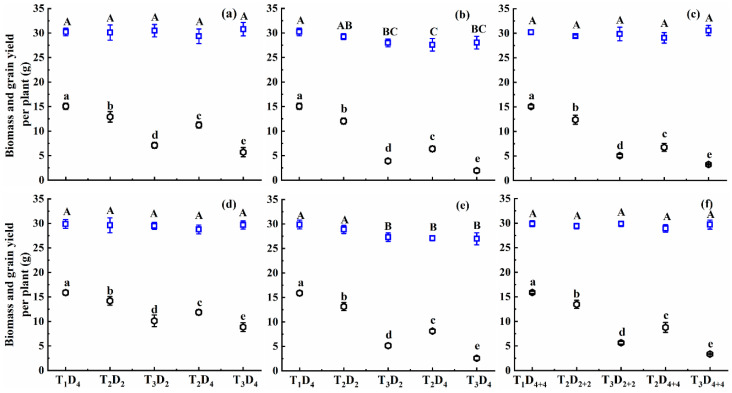
Biomass per plant at maturity (BPP_M_) and grain yield per plant (YPP) under different high-temperature treatments at booting, flowering, and combined stages during the 2016–2017 growing seasons. (**a**) Huaidao-5 at booting treatment; (**b**) Huaidao-5 at flowering treatment; (**c**) Huaidao-5 at combined stages treatment; (**d**) Wuyunjing-24 at booting treatment; (**e**) Wuyunjing-24 at flowering treatment; (**f**) Wuyunjing-24 at combined stages treatment. □ indicates BPP_M_ and ⬡ indicates YPP. Different letters indicate significant differences at the 0.05 level. Vertical bars represent the standard deviation of the mean.

As shown in Figure 5, the high-temperature effect on aboveground biomass per plant at maturity (BPP_M_) showed different trends for different stages, cultivars, and temperature levels and durations. Compared with T_1_D_4_, a decline in BPP_M_ was observed under temperature level T_2_ but revealed different results for T_3_, and for Huaidao-5 showed an increase of 1.8% and 1.1% at booting and combined stages, respectively. Compared with T_1_D_4_, the decline in BPP_M_ was more obvious at flowering (3.3–7.3% and 3.3–9.6% for Huaidao-5 and Wuyunjing-24 for T_2_ and T_3_, respectively) than at other stages. In addition, high-temperature treatment at booting, flowering, and combined stages significantly decreased the YPP of both cultivars. The decrease in YPP was higher at the flowering stage, followed by combined and booting stages. The decreases in YPP for T_2_ and T_3_ compared with T_1_D_4_ at booting, flowering, and combined stages were 14.3–62.2%, 20.2–87.1%, and 17.7–78.3% for Huaidao-5, and 10.6–44.3%, 17.3–83.8%, and 15.2–79.0% for Wuyunjing-24, respectively. BPP_M_ did not show a uniform pattern at booting and combined stages because there was an increase in BPP_M_ under T_3_ but an obvious decrease for T_2_, particularly for Huaidao-5. However, BPP_M_ showed a significant decline at flowering, and for each 1 °C increase in high temperature, BPP_M_ decreased by 0.6% and 0.7% for Huaidao-5 and 0.7% and 0.9% for Wuyunjing-24 under D_2_ and D_4_, respectively. Additionally, YPP decreased with increasing temperature levels for both cultivars. For an increase of 1 °C in high temperature at booting, flowering, and combined stages, the YPP decreased by 4.0%, 5.7%, and 5.1%, and 4.9%, 7.3% and 6.6% for Huaidao-5, and decreased by 2.8%, 5.1%, and 4.9%, and 3.6%, 6.8%, and 6.4% for Wuyunjing-24 for 2 and 4 days’ duration for individual stages and 4 and 8 days’ duration for combined stages, respectively. This pattern indicates that Huaidao-5 was more affected by high temperature than Wuyunjing-24 (Figure 5).

At the same high-temperature level, BPP_M_ decreased under T_2_ whereas it increased under T_3_, except for the flowering stage. Under T_2_, BPP_M_ declined by 1.3%, 2.7%, and 0.3% for Huaidao-5, whereas for Wuyunjing-24, it decreased by 1.4%, 3.0%, and 0.4% for each one-day increase in high-temperature duration at booting, flowering, and combined stages. However, for each one-day increase in high-temperature under T_3_, BPP_M_ showed an increase of 0.5% and 0.6% for Huaidao-5 and 0.3% and 0.1% for Wuyunjing-24 at booting and combined stages, respectively, whereas, at flowering, T_3_ showed a decrease of 0.5% for Wuyunjing-24. Moreover, at the same high-temperature level, YPP decreased with increasing high-temperature duration for both cultivars at booting, flowering, and combined stages. The YPP was more sensitive to high temperature for Huaidao-5 than Wuyunjing-24. YPP declined by 5.5%, 18.7%, and 9.3% under T_2_, and 4.7%, 6.5%, and 3.0% under T_3_ for Huaidao-5, whereas for Wuyunjing-24, it decreased by 7.3%, 15.8%, and 7.4% under T_2_, and 4.0%, 8.1%, and 3.6% under T_3_, for each one-day increase in high-temperature duration at booting, flowering, and combined stages, respectively (Figure 5). Increasing temperature level, duration, and their interaction showed a significant decrease in YPP at all growth stages (*p* < 0.05), whereas BPP_M_ showed a significant variation only under high-temperature levels at the flowering stage (Table 1).

### 2.2. Quantifying the Effects of Extreme High-Temperature Stress on Photosynthetic Property Parameters, Biomass Accumulation, and Grain Yield in Response to HDD

**Figure 6:**
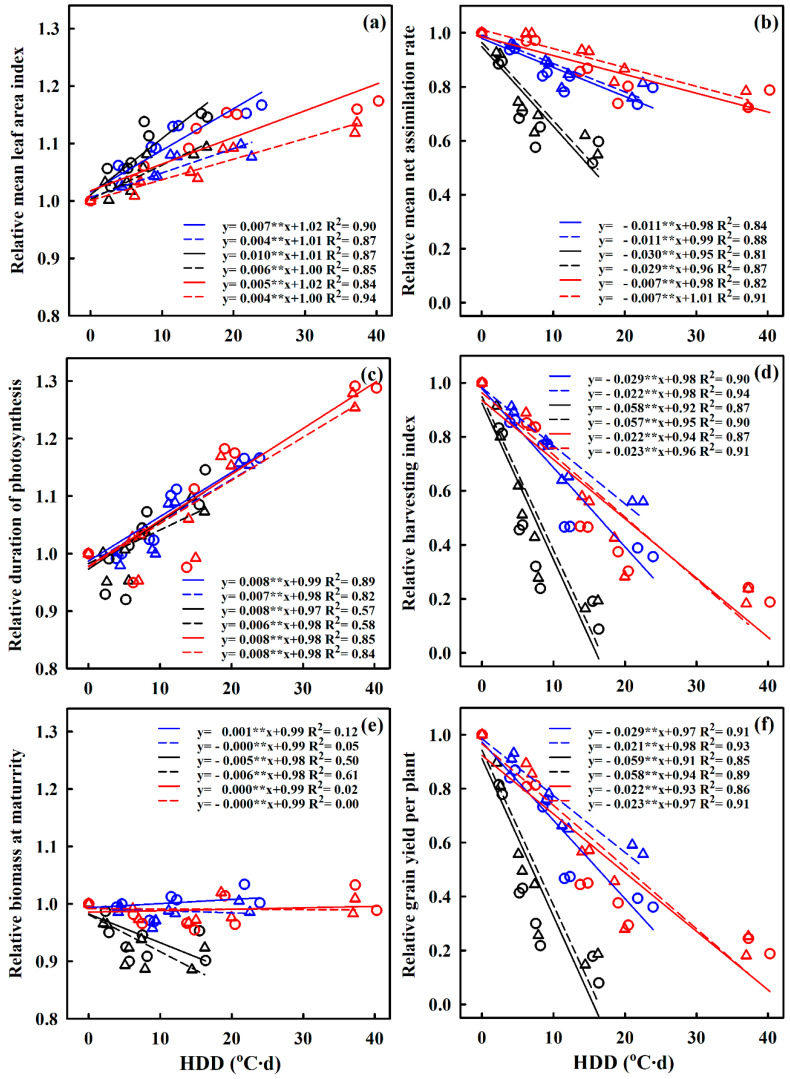
Relationships between the relative grain yield per plant (YPP), biomass accumulation at maturity (BPP_M_), harvest index (HI), mean leaf area index (MLAI), mean net assimilation rate (MNAR), and duration of photosynthesis (DOP) in rice and heat degree days (HDD) at booting, during the 2016–2017 growing seasons. (**a**) Relationship between MLAI and HDD; (**b**) relationship between MNAR and HDD; (**c**) relationship between DOP and HDD; (**d**) relationship between HI and HDD; (**e**) relationship between BPP_M_ and HDD; (**f**) relationship between YPP and HDD. ○ and the solid line indicates Huaidao-5, ∆ and the dashed line indicates Wuyunjing-24; Blue, black, and red lines are for booting, flowering, and combined stages, respectively. ** *p* < 0.01.

The relationships between HDD and MLAI, MNAR, DOP, HI, BPP_M_, and YPP were investigated to quantify the effects of high temperature at booting, flowering, and combined stages on photosynthetic property parameters, biomass accumulation, and grain yield. The HDD–MLAI and HDD–DOP showed significantly positive (*p* < 0.05) relationships, whereas the relationships between HDD–MNAR, HDD–HI, HDD–YPP, and HDD-BPP_M_ were significantly negative, except for Huaidao-5, for which HDD-BPP_M_ showed a positive relationship at booting and combined stages due to the increase in BPP_M_ under T_3_ (Figure 6). For each 1 °C d increase in HDD, MLAI and DOP increased by 0.7% and 0.8%, 1.0% and 0.8%, and 0.5% and 0.8% for Huaidao-5, and 0.4% and 0.7%, 0.6% and 0.6%, and 0.4% and 0.8% for Wuyunjing-24 at booting, flowering, and combined stages, respectively. Results revealed that Huaidao-5 experienced a greater increase in MLAI and DOP compared to Wuyunjing-24. Moreover, MNAR, HI, and YPP at booting, flowering, and combined stages decreased by 1.1%, 2.9%, and 2.9%; 3.0%, 5.8%, and 5.9%; and 0.7%, 2.2%, and 2.2% for Huaidao-5, and 1.1%, 2.2% and 2.1%; 2.9%, 5.7%, and 5.8%; and 0.7%, 2.3%, and 2.3% for Wuyunjing-24 for each 1 °C d increase in HDD. Additionally, flowering was the most sensitive stage to high temperature for MNAR, HI, and YPP. Furthermore, BPP_M_ showed a different pattern compared to the other indices; overall, it showed no significant difference, but at flowering, a decrease of 0.5% and 0.6% for Huaidao-5 and Wuyunjing-24 was observed, respectively.

### 2.3. Relationships Between Grain Yield, Biomass Accumulation and Photosynthetic Property Parameters under High-Temperature Condition

**Figure 7:**
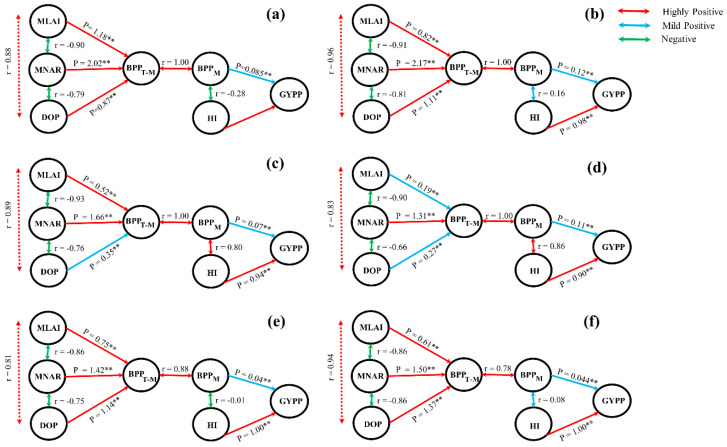
Path coefficient diagrams showing the interrelationships among grain yield per plant (YPP), biomass accumulation per plant at maturity (BPP_M_), harvest index (HI), biomass accumulation per plant from the first day of treatment to maturity (BPP_T-M_), mean leaf area index (MLAI), mean net assimilation rate (MNAR), and duration of photosynthesis (DOP) under high temperature at booting, flowering, and combined stages during the 2016–2017 growing seasons. In the diagram, single-headed arrows indicate path coefficients (P), and double-headed arrows indicate simple linear correlation coefficients (r). (**a**) Huaidao-5 at booting treatment; (**b**) Wuyunjing-24 at booting treatment; (**c**) Huaidao-5 at flowering treatment; (**d**) Wuyunjing-24 at flowering treatment; (**e**) Huaidao-5 at combined treatment; (**f**) Wuyunjing-24 at combined treatment. NS: not significant, * *p* < 0.05 and ** *p* < 0.01.

Path analysis was used to quantify the contributions of MLAI, MNAR, and DOP to BPP_T-M_ and the importance of HI and BPP_M_ for YPP. MNAR was the principle explanatory variable under high-temperature treatment, making a greater contribution to BPP_T-M_ (Figure 7). Values of P for MNAR were higher at booting stages, followed by combined and flowering. In addition, DOP made a greater contribution to BPP_T-M_ at combined stages, followed by booting and flowering. MLAI also made a greater contribution to combined stages followed by booting and flowering. The relationships between MLAI and DOP were significantly positive, whereas the relationships between MLAI and MNAR and between MNAR and DOP were significantly negative. Path analysis was carried out to quantify the contribution of HI and BPP_M_ to YPP. Results showed that under high-temperature treatments, HI contributed more to YPP than to BPP_M_. However, a significant positive correlation was observed between HI and BPP_M_ for both cultivars, except Huaidao-5 at booting and combined stages, which showed a slight increase in BPP_M_ compared to T_1_, resulting in a negative correlation between HI and BPP_M_.

## 3. Discussion

### 3.1. Biomass Production and Grain Yield Responses to High Temperatures

High-temperature stress is one of the major environmental factors leading to yield losses as a consequence of restricted plant growth and photosynthetic production. The impacts of high-temperature stress on biomass accumulation and yield formation in rice are not only reliant on the high-temperature level, duration, and growth stage but also the frequency of its incidence. Subjecting plants to high-temperature stress twice (pre-exposure to a sub-lethal high temperature at vegetative and early reproductive stages, accompanied by severe lethal treatment at the flowering stage) can improve the heat resistance of rice and may partially compensate for the adverse effects of high temperature [34]. Consequently, yield, biomass accumulation, and photosynthetic property parameters under heat stress at booting, flowering, and combined (booting + flowering) stages were analyzed to compare the effects of single-stage heat stress with combined stages of heat stress. This study illustrated that YPP was substantially reduced under increasing temperature levels, durations, and their interactions, despite the non-significant effect of high temperature on biomass accumulation as reported in previous studies on single-stage heat stress [11,36,38]. However, parameters such as mean leaf area index (MLAI), mean net assimilation rate (MNAR), and duration of photosynthesis (DOP), which determine the biomass accumulation, were significantly affected under heat stress treatments [23,25].

In this study, increased DOP under T_3_ was attributed to the reduced sink capacity, which resulted in limiting the translocation of nutrients to vegetative organs due to its incapability to store assimilates as a consequence of increased photosynthetic productivity. Due to this reduced sink capacity, the number of green leaves increased after grain filling under high-temperature treatments, especially under T_3_ compared to T_1_, and delayed the senescence of leaves and resulted in increased MLAI. Short-term heat stress at the booting stage reduced sink size, resulting in increased photosynthetic rate and leaf area at maturity [12]. Thus, our results reveal that increasing MLAI under high temperature has the potential to compensate for biomass accumulation, which is consistent with previous research on rice [11,27]. Due to dual heat stress during combined stages, the increase in MLAI and DOP were higher at combined stages, followed by booting and flowering stages. In addition, under heat stress, Huaidao-5 showed a greater increase in MLAI and DOP than Wuyunjing-24. The contribution of MLAI and DOP to BPP_T-M_ was lowest at flowering, which indicates that MLAI and DOP under high-temperature were not affected at booting and combined stages.

The decline in MNAR was greater at flowering, followed by combined and booting stages, and Huaidao-5 was more vulnerable to heat stress compared to Wuyunjing-24. Flowering was the most affected stage because heat stress caused more fertility and yield loss, which results in a greater sink loss at the flowering stage compared to other stages [8,36], and, ultimately, a decline in MNAR. These results show that multiple heat stress compensated for the injuries caused by heat stress compared to single-stage heat stress. High-temperature pre-treatment activates the subcellular antioxidant system to suppress oxidation bursts in photosynthesis equipment, thereby enhancing the ability to withstand subsequent high-temperature stress in plants; this is consistent with our results [33]. MNAR contributed more to BPP_T-M_ than MLAI and DOP, and this contribution was greater at the booting stage than at the other stages. These findings are in line with previous studies [39,40], in which MNAR contributed more significantly to crop growth rate than other factors. The identical trend of a decrease in MNAR and YPP indicated a strong relationship between YPP and MNAR. These results also indicate that improving MNAR could compensate for final grain yield loss [9,28]. The negative correlation between photosynthetic efficiency (MNAR) and photosynthetic area (MLAI) was also consistent with previous reports [25,41].

Our results further indicate that DOP increased under extremely high-temperature T_3_ but showed different patterns for different durations, stages, and cultivars under T_2_. GDD decreased under T_2_ due to the early maturity of rice plants, but the addition of HDD during treatment increased DOP for some treatments, especially for D_4_ and combined stages. Some previous studies also showed a decrease in DOP similar to that of the T_2_ treatments in this study [4,42]. According to Kim et al. (2011), early maturity under high temperature was due to rapid grain filling and reduced grain-filling duration. Conversely, short-term heat stress under extremely high-temperature T_3_ almost destroyed the panicle and resulted in a loss of sink capacity, and delayed the DOP [11,12]. The above results imply that after the loss of sink, the leaves were still maintaining photosynthetic capacity and were supplying the assimilates to other plant tissues, except for grain. Due to the delay in the senescence process, MLAI and DOP were increased more by the heat stress at combined stages. Previous research showed that, compared with single-stage heat stress, multiple heat stresses might cause down-regulation of bifunctional nuclease I (enzyme), which is involved in programmed cell death and senescence [43], thus verifying our results.

Additionally, assimilates were translocated to the axillary buds and roots and generated new young panicle-bearing tillers from the axillary buds as a consequence of limited sink capacity [44]. Consequently, the final harvesting panicles under T_3_ treatment have both original and regenerated tillers. Therefore, short-term heat stress during booting and combined stages did not cause any significant change in final BPP_M_. Conversely, BPP_M_ slightly increased under T_3_ at booting and combined stages due to the production of a massive quantity of regenerating tillers compared with T_1_. This increase in BPP_M_ is also in line with [12], in which very high temperature (maximum/minimum/mean, 44/34/39 °C) caused a significant increase in BPP_M_. However, there was a decrease in BPP_M_ under flowering treatments compared with T_1_ because the production of regenerating tillers at flowering was lower than that at booting and combined stages (Figure S5), which is also consistent with previous reports [11,45].

The current study illustrated that YPP substantially declined with increasing temperature levels, durations, and their interactions, which is consistent with previous studies of heat stress at a single stage [11,12,36,37]. Despite the dual heat stresses at combined stages compared to the flowering stage, the decline of YPP was less than that at flowering, which revealed that the combined stages alleviated the injuries caused by heat stress. Previous studies also demonstrated that heat-acclimatized plants produce a higher yield due to better signaling pathways, enhanced stem reserve remobilization to grain, better activities of antioxidant enzymes, enhanced ROS scavenging, greater photosynthetic capacity, stomatal conductance, and chlorophyll content in comparison to non-heat-acclimatized plants [33,35,46]. According to this study, Huaidao-5 was more affected by high temperature than Wuyunjing-24. Additionally, the heat tolerance of Wuyunjing-24 has also been reported in earlier studies [12,15]. So, these results are in line with [34], which suggested that the strategy of mitigating heat stress could be more prevalent in the tolerant genetic background such as Wuyunjing-24 in this study than in sensitive backgrounds.

Additionally, path analysis revealed that HI and BPP_M_ both contributed significantly to YPP, but a greater reduction in yield was caused by HI compared to BPP_M_. These findings reveal HI is more important for YPP compared to BPP_M_ under high temperature. Environmental factors determine photosynthetic efficiency, and it is clear that trade-offs between photosynthetic duration and photosynthetic efficiency can occur. In addition, negative correlations between leaf area and photosynthetic efficiency [47], leaf area duration, and maximum photosynthetic efficiency [23,41] have been reported for a range of crops. The literature described above is also in line with our findings, in which a significant negative correlation was observed between MLAI and MNAR and between MNAR and DOP.

### 3.2. Relationships of HDD with Relative Biomass Production and Grain Yield

Heat degree days (HDD) comprehensively integrates the duration and intensity of high temperatures. In addition, HDD quantitatively explains the effects of high-temperature events occurring in different years and locations on crop yield [15,16]. The current study showed significant negative correlations of MNAR, HI, and YPP with HDD for both cultivars under high-temperature treatments, which is also consistent with previous literature [4,11,26,27]. However, significant positive correlations of MLAI and DOP with HDD were observed. These results reveal that HDD had a greater negative effect on MNAR, HI, and YPP at flowering than other stages, and Huaidao-5 was the more heat-susceptible cultivar compared to Wuyunjing-24. However, under heat stress at combined stages, HI and YPP of Wuyunjing-24 showed a greater decline, whereas Huaidao-5 was more affected by single-stage heat stress. These results indicate that Wuyunjing-24 showed greater heat tolerance under a single heat stress, whereas Huaidao-5 showed more heat tolerance of multiple heat stresses. These results are consistent with a previous study, which showed that one rice cultivar has better basal thermo-tolerance, whereas the other has a higher capacity for heat-stress memory [48]. In addition, a negative correlation between BPP_M_ and HDD was observed, except for Huaidao-5 at booting and combined stages, for which the correlation between BPP_M_ and HDD was positive [12].

### 3.3. Limitation of this Study

Although appropriate management practices were undertaken during the experiment, rice plants still had some limitations, especially for the expansion of roots in pots [11,49]. Variations in crop production arise in the field due to the microclimate, soil properties, and developmental variations between the main stems and tillers. In comparison, crop responses under controlled conditions vary from those under field conditions, which are also subject to multiple abiotic stresses, such as low radiation, heat waves, high winds, and different other stress interactions. The results reflect the effect of high temperature on biomass accumulation and yield formation to a certain extent and can help in the improvement of crop growth models and screening of heat-resistant rice cultivars. To reexamine these results, both physiological and deep molecular studies, under conditions of short-term extreme temperature during reproductive stages, are needed.

## 4. Materials and Methods

### 4.1. Experimental Design

**Figure 8:**
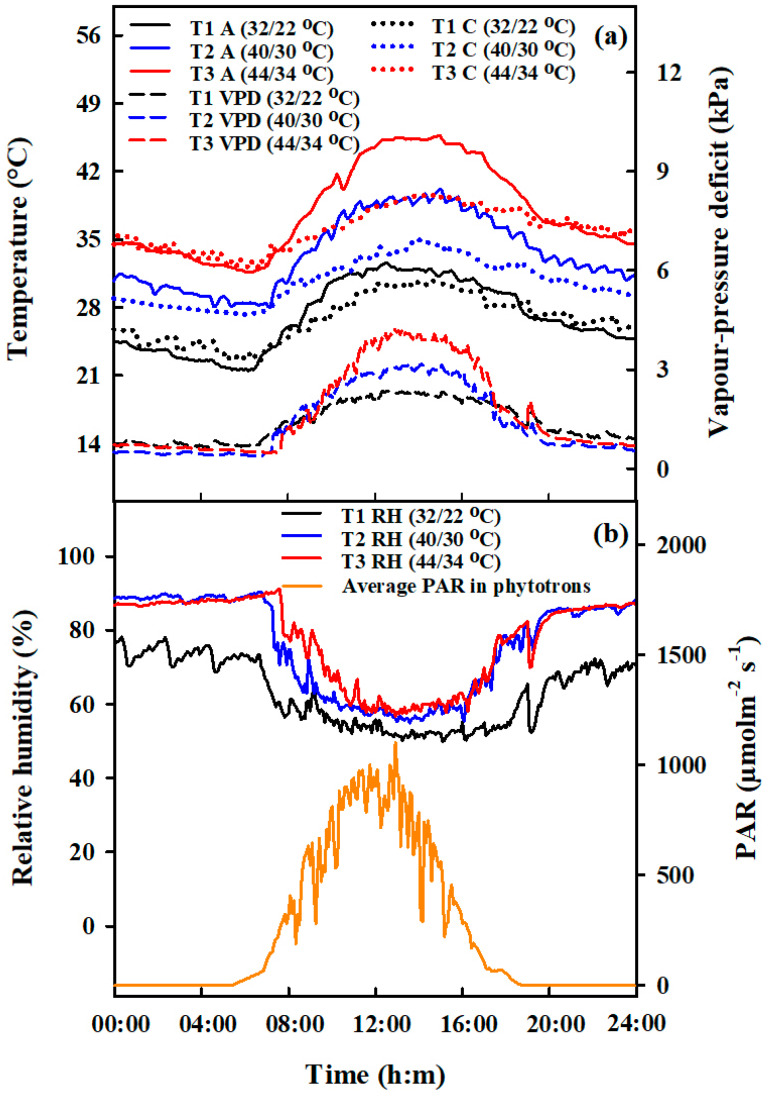
Diurnal variations of air (A, °C, solid lines, (**a**)—left vertical axis), and canopy temperature (C, °C, dotted lines, (**a**)—left vertical axis), vapor pressure deficit (VPD, kPa, dashed lines, (**a**)—right vertical axis), relative air humidity (RH, %, solid lines, (**b**)—left vertical axis) and average photosynthetically active radiation (PAR, μmolm^−2^ s^−1^, (**b**)—right vertical axis) in phytotrons during heat stress treatments in 2016.

A two-year environment-controlled phytotrons experiment was conducted at Rugao (120.33° E, 32.23° N) located in Jiangsu Province, China, during 2016–2017. Two Japonica cultivars were used: Huaidao-5 (heat-sensitive cultivar, bred and approved by Huaian Institute of Agricultural Sciences, Huaian, China in 2000) and Wuyunjing-24 (heat-tolerant cultivar, bred and released by Wujin Agriculture Institute, Changzhou, China in 2010). Transplantation of seedlings was performed at the three-leaf stage in plastic pots filled with 22.4 L soil with a diameter, height, and volume of 35.6 cm, 29.8 cm, and 25.0 L, respectively. The pots were flooded until one week before harvesting with a planting density of 3 hills per pot (2 seedlings per hill). Eleven pots per m^2^ were arranged together, which was equivalent to a planting density of 66 plants per m^2^ and was compatible with that of Japonica rice (60 to 75 plants per m^2^) grown under field conditions in the region (Jiangsu Province Commission of Agriculture, 2011). The basal fertilizer was applied at the rate of N (1.5 g), P_2_O_5_ (1.5 g), and K_2_O (2 g) per plot before transplanting. Supplemental N at the rate of 0.3 g N and 1.2 g N per pot was top-dressed at mid-tillering and panicle initiation stages, respectively. To protect from biotic and abiotic stresses, irrigation, weeding, and pest and disease control were undertaken following local standards of rice cultivation. Heat stress was the only limiting factor in this study. Rice plants were kept under ambient conditions before and after high-temperature stress. During the rice-growing season (from May to October), the ambient maximum and minimum daily temperatures averaged 28.3 and 20.1 °C, respectively (with an average daily temperature of 24.2 °C) (Figure S6). Pots with plants having homogenous tiller number were moved into phytotron chambers (L × W × H; 3.4 × 3.2 × 2.8 m) and divided into three sets for heat-stress treatments at the target development stages. Pots were moved to phytotrons for high-temperature stress when rice plants reached the booting stage (50% of plants have swelling of the flag leaf sheath, and panicle becomes visible to the naked eye having growth of about 2 mm in length) and flowering (at 50% flowering of panicles). The initiation time of the first spikelet flowering was recorded, and the flowering date was determined when 50% of panicles per pot initiated flowering. Moreover, for combined stages, the same sets of rice plants treated with heat stress at the booting stage were again transferred into phytotrons when they reached flowering. High-temperature stress at booting, flowering, and combined stages is shown in Figure 8.

Previous research has shown that the daily optimal average temperature for local rice plants during booting and flowering stages was 23–26 °C (with maximum and minimum temperatures of 29–32 °C and 17–21 °C, respectively) [11,12]. Thus, the target daily minimum and maximum temperatures (Tmin/ Tmax) for three treatments were set as 22/32 °C (T_1_), 30/40 °C (T_2_), and 34/44 °C (T_3_) with two durations, 2 days (D_2_) and 4 days (D_4_) (Table 2). T_1_ (which was considered to be the optimum temperature for rice growth during the booting and flowering stages) was only treated for 4 days and was regarded as the control treatment [11,12]. However, for combined stages, heat-stress durations were 4 days (D_2+2_) and 8 days (D_4+4_) because, during combined heat stress, the same sets of rice plants were treated twice, first at booting, followed by flowering heat stress. Air temperature (Ta, °C), soil temperature (Ts, °C), relative humidity (RH, %), and photosynthetically active radiation (PAR, μmolm^−2^ s^−1^) were measured in the phytotrons using a 5TM sensor (METER Group, Inc., Washington, USA), VP-3 sensor (METER Group, Inc., Washington, USA), and PYR solar radiation sensor (METER Group, Inc., Washington, USA), respectively. Following the design of the local environment, temperature and RH in the phytotrons were fixed and controlled for every hour. Vapor pressure deficit (VPD) was calculated according to FAO standards [50]. An infrared radiometer (SI-111, Apogee Instruments, Logan, Utah, USA) was used to monitor canopy temperature (Tc, °C) and placed 1.6 m above ground at the center of the chamber facing diagonally downward to an area of 0.8 m^2^ of plant surface. Ta, Tc, VPD, RH, and PAR were regularly recorded, as demonstrated in Figure 8.

### 4.2. Leaf Area and Biomass Measurements

After high-temperature treatment, plant samples (Total 54 samples for each sampling, as there were 3 temperature levels, 2 durations, 3 growth stages, and 3 replications for every treatment) were collected at the interval of 5–7 days during the growing season until maturity after heat stress treatment and the green leaf area was measured to calculate LAI with the help of an LAI-3000 portable area meter (LI-COR, Lincoln, NE, USA). Three replications (i.e., three pots) across all of the treatments were measured at each sampling date during 2016 and 2017. Sampled plants were separated into green leaves, dead leaves (leaf color ≥ 80% yellow), stems, sheaths, and panicles. Plant organs were oven-dried at 105 °C for 30 min, then at 80 °C to obtain a constant weight to determine the dry matter weight of different organs. The measured LAI and aboveground dry matter per plant under different high-temperature treatments are shown in Figures S1–S4.

### 4.3. Measurement of Grain Yield

After maturity, five pots of rice were randomly picked from each treatment, each with three replications (15 pots in total) to evaluate grain yield per plant (YPP). High temperature inhibited the translocation of assimilates toward panicles under T_2_ and T_3_ treatments, causing carbon and nitrogen to be transported to the vegetative parts. As a result, the final panicles under the T_2_ and T_3_ treatments had original and regenerated tillers [44]. Therefore, a high-temperature effect on grain yield was observed for original and total tillers (original + regenerated tillers).

### 4.4. Calculation of Grain Yield Formation

According to [23,25], the grain yield can be expressed as follows (Equations (1)–(3)):

(1) $$\mathrm{Grain}\;\mathrm{yield}={\mathrm{BPP}}_{M}\times \mathrm{HI}\;$$

(2) $${\mathrm{BPP}}_{M}={\mathrm{BPP}}_{B}+{\mathrm{BPP}}_{T-M}\;$$

(3) $${\mathrm{BPP}}_{T-M}=\mathrm{MLAI}\;\times \;\mathrm{MNAR}\;\times \;\mathrm{LAD}\;$$

BPP_M_ is the biomass per plant at maturity calculated by Equation (2), where BPP_B_ is BPP before treatment and BPP_T-M_ is the accumulated aboveground biomass measured from the first day of high-temperature treatment until maturity, and HI is the harvest index for each treatment. MLAI and MNAR are mean leaf area index and mean net assimilation rate calculated by Equations (4)–(6) and by Equation (7), respectively. LAD is the accumulated photosynthetic leaf area per unit land area from high-temperature treatment to maturity and calculated from Equation (5) using an integral calculator.

(4) $$\mathrm{MLAI}=\frac{\mathrm{LAD}}{\mathrm{DOP}}=\frac{\mathrm{LAD}}{{\mathrm{GDD}}_{m}-{\;\mathrm{GDD}}_{i}\;}\;$$

(5) $$\mathrm{LAD}=\int_{{\mathrm{GDD}}_{i}}^{{\mathrm{GDD}}_{m}}\mathrm{LAI}\;$$

(6) $$\mathrm{LAI}=a+\mathrm{bGDD}+{\mathrm{cGDD}}^{2}+{\mathrm{dGDD}}^{3}$$

(7) $$\mathrm{MNAR}=\frac{{\mathrm{BPP}}_{T-M}}{\mathrm{LAD}}\;$$

In Equation (4), DOP is the duration of photosynthesis and was calculated from growing degree days (GDD) from the first day of high-temperature treatment (GDD_i_) to maturity (GDD_m_), using Equations (8–10). LAI is the measured leaf area index using a cubic curve to fit with GDD. GDD is the sum of the daily growing degree days. DDi is the average of the hourly growing degree days on the ith day after high-temperature treatment, and DDt is the hourly growing degree days at the *t*th hour of the day. T_b_ (°C) represents the base temperature and was set to 10 °C [51], and T_t_ (°C) is the hourly air temperature of the *t*th hour of a day and was recorded by EM50 data loggers in the phytotrons and outside field.

(8) $$\mathrm{GDD}=\overset{m}{\underset{i=1}{\sum }}{\mathrm{DD}}_{i}$$

(9) $$\mathrm{DDi}=\frac{1}{24}\overset{24}{\underset{t=1}{\sum }}{\mathrm{DD}}_{t}\;$$

(10) $${\mathrm{DD}}_{t}=\left(\begin{array}{c} 0,{\;T}_{t}<T_{b}\\ T_{t}-T_{b},{\;T}_{t}\;\geq T_{b} \end{array}\right)$$

### 4.5. Statistical Analysis

Experimental data were analyzed using a mixed linear model in R 3.3.0 software, and analysis of variance (ANOVA) was performed using SPSS 18.0 statistical software (SPSS Inc., Chicago, IL, USA) to estimate the effects of high-temperature levels, duration, and their interactions on grain yield (YPP), BPP_M_, HI, MLAI, MNAR, and DOP. Duncan’s new multiple range test was used to analyze the differences among treatments at 0.05 significance levels. Relationships between heat degree days (HDD) and agronomic traits were established using simple linear regression to quantify the general effects of high-temperature levels and durations on agronomic traits (YPP, BPP, HI, MLAI, MNAR, and DOP). HDD was applied to quantify the combined effects of heat stress severity and duration [16]. In addition, the contribution of BPP_M_ and HI to YPP and the contribution of MLAI, MNAR, and DOP to BPP_T-M_ was determined by path coefficient analysis (P) of SPSS 18.0.

## 5. Conclusions

Rice plants pre-exposed to heat stress at the booting stage recalled a heat-stress memory that activated stress-scavenging mechanisms during heat stress at the flowering stage. Functional growth analysis was used to determine the relative importance of photosynthetic duration in determining biomass production. Loss of grain yield during heat stress at booting, flowering, and combined stages was not restricted by photosynthesis. In contrast, the developing panicles could not assimilate the excessive photosynthetic products due to the lost sink capacity. Thus, these photosynthetic assimilates moved toward vegetative parts. As a result, short-term heat stress during the reproductive stages had little impact on final biomass accumulation. Plants under heat stress at combined stages significantly compensated for damage to the mean leaf area index (MNAR), harvest index (HI), and grain yield per plant (YPP) by increasing the duration of photosynthesis (DOP), resulting in an increase in mean leaf area index and biomass per plant at maturity (BPP_M_) especially for treatment T_3_. Thus, knowledge about pre-treatment with a lethal temperature, followed by lethal temperature adaptation and acclimation in rice cultivars, offers novel perspectives for understanding how crop performance can be enhanced under changing climate conditions. These results could help to evaluate and predict rice yield from biomass accumulation in future scenarios of heat events due to climate change. The results may also help to mitigate susceptibility to high temperature and improve algorithms in rice models under high-temperature conditions.

## Figures and Tables

**Table 1:** Probability values of the main effects of high-temperature level, duration, and their interaction on mean leaf area index (MLAI), mean net assimilation rate (MNAR), duration of photosynthesis (DOP), harvest index (HI), biomass per plant at maturity (BPP_M_), and grain yield per plant (YPP) under the treatments of high temperature at booting, flowering, and combined stages during the 2016–2017 growing seasons.

Stage	Cultivar	Photosynthetic Properties	T	D	T × D
Booting	Huaidao-5	MLAI	<0.0001	ns	ns
		MNAR	<0.0001	ns	ns
		DOP	0.011	ns	ns
		HI	<0.0001	0.002	ns
		BBP_M_	ns	ns	ns
		GYPP	<0.0001	0.001	0.034
	Wuyunjing-24	MLAI	0.002	ns	ns
		MNAR	<0.0001	ns	ns
		DOP	0.045	ns	ns
		HI	<0.0001	<0.0001	0.012
		BBP_M_	ns	ns	ns
		GYPP	<0.0001	<0.0001	0.009
Flowering	Huaidao-5	MLAI	<0.0001	ns	ns
		MNAR	<0.0001	0.019	0.046
		DOP	0.002	ns	ns
		HI	<0.0001	<0.0001	<0.0001
		BBP_M_	<0.0001	ns	ns
		GYPP	<0.0001	<0.0001	<0.0001
	Wuyunjing-24	MLAI	0.003	ns	ns
		MNAR	<0.0001	0.004	0.048
		DOP	ns	ns	ns
		HI	<0.0001	<0.0001	<0.0001
		BBP_M_	<0.0001	0.029	ns
		GYPP	<0.0001	<0.0001	<0.0001
Combined	Huaidao-5	MLAI	<0.0001	ns	ns
		MNAR	<0.0001	ns	ns
		DOP	0.010	ns	ns
		HI	<0.0001	<0.0001	<0.0001
		BBP_M_	ns	ns	ns
		GYPP	<0.0001	<0.0001	<0.0001
	Wuyunjing-24	MLAI	0.001	ns	ns
		MNAR	<0.0001	ns	ns
		DOP	0.004	ns	ns
		HI	<0.0001	<0.0001	<0.0001
		BBP_M_	ns	ns	ns
		GYPP	<0.0001	<0.0001	<0.0001

Numbers in the table indicate the *p* values of main and interaction effects for which at least one variable was detected as significant (*p* ≤ 0.05); ns: not significant (*p* > 0.05).

**Table 2:** Summary of high-temperature stress treatments in phytotron experiments.

Cultivar	Temperature Levels	Stage	Durations
Huaidao-5 and Wuyunjing-24	T_1_(32/22 °C) T_2_(40/30 °C) T_3_(44/34 °C)	Booting	D_2_ (two days booting) and D_4_ (four days booting)
		Flowering	D_2_ (two days flowering) and D_4_ (four days flowering)
		Combined stages	D_2+2_ (four days = 2 days booting + 2 days flowering) and D_4+4_ (eight days = 4 days booting + 4 days flowering)

## Data Availability

All data generated or analyzed during this study are included in this article.

## Supplementary Materials

The following are available online at https://www.mdpi.com/article/10.3390/plants10051021/s1, Figure S1: The measured leaf area index (LAI) under different high-temperature treatments at booting, flowering, and combined stages in 2016, Figure S2: The measured leaf area index (LAI) under different high-temperature treatments at booting, flowering, and combined stages in 2017, Figure S3: The measured aboveground biomass per plant (g) under different high-temperature treatments at booting, flowering, and combined stages in 2016, Figure S4: The measured aboveground biomass per plant (g) under different high-temperature treatments at booting, flowering, and combined stages in 2017, Figure S5: Grain yield per plant including original and regenerated tillers under different high-temperature treatments at booting, flowering, and combined stages during the 2016–2017 growing seasons, Figure S6: Daily (a) maximum and (b) minimum temperatures of the natural environment from May to October during the rice growing seasons 2016–2017.
