# Supplementary material for: Individual and Combined Effects of Booting and Flowering High-Temperature Stress on Rice Biomass Accumulation
Source: Plants (Basel). 2021 May 20;10(5):1021. doi: 10.3390/plants10051021 (PMC8160744; doi:10.3390/plants10051021)
Supplement: Supplementary file 1 [file plants-10-01021-s001.zip › plants-1202862-supplementary.pdf]

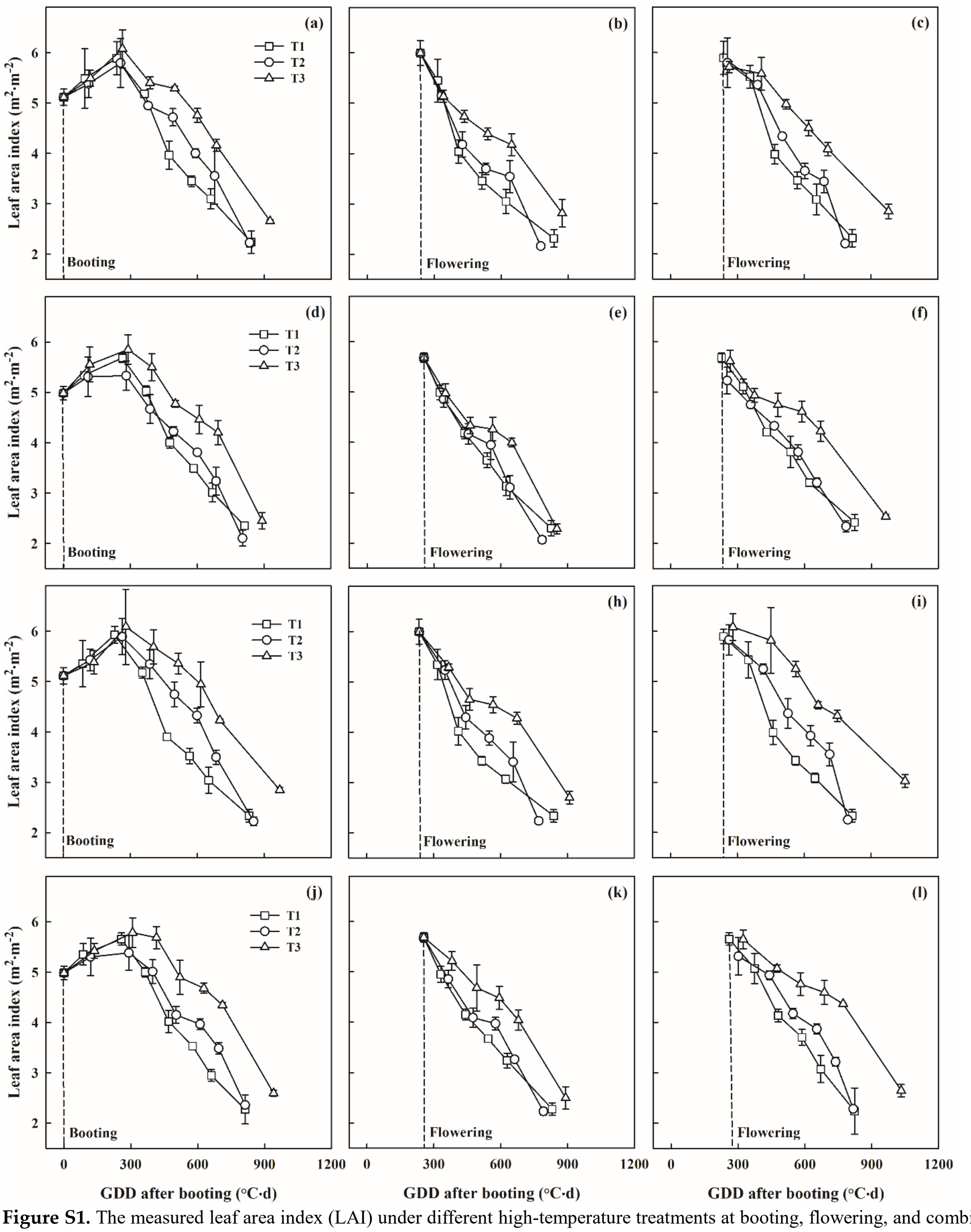

Figure S1. The measured leaf area index (LAI) under different high-temperature treatments at booting, flowering, and combined stages in 2016. (a-c): Huaidao-5 under 2 days' duration (D_2) at booting and flowering stages and under 4 days (D_{2+2}) at combined stages, respectively; (d-f): Wuyunjing-24 under 2 days' duration (D_2) at booting and flowering stages and under 4 days (D_{2+2}) at combined stages, respectively; (g-i): Huaidao-5 under 4 days' duration (D_4) at booting and flowering stages and under 8 days (D_{4+4}) at combined stages, respectively; (j-l): Wuyunjing-24 under 4 days' duration (D_4) at booting and flowering stages and under 8 days (D_{4+4}) at combined stages. Vertical bars represent standard deviation of mean.

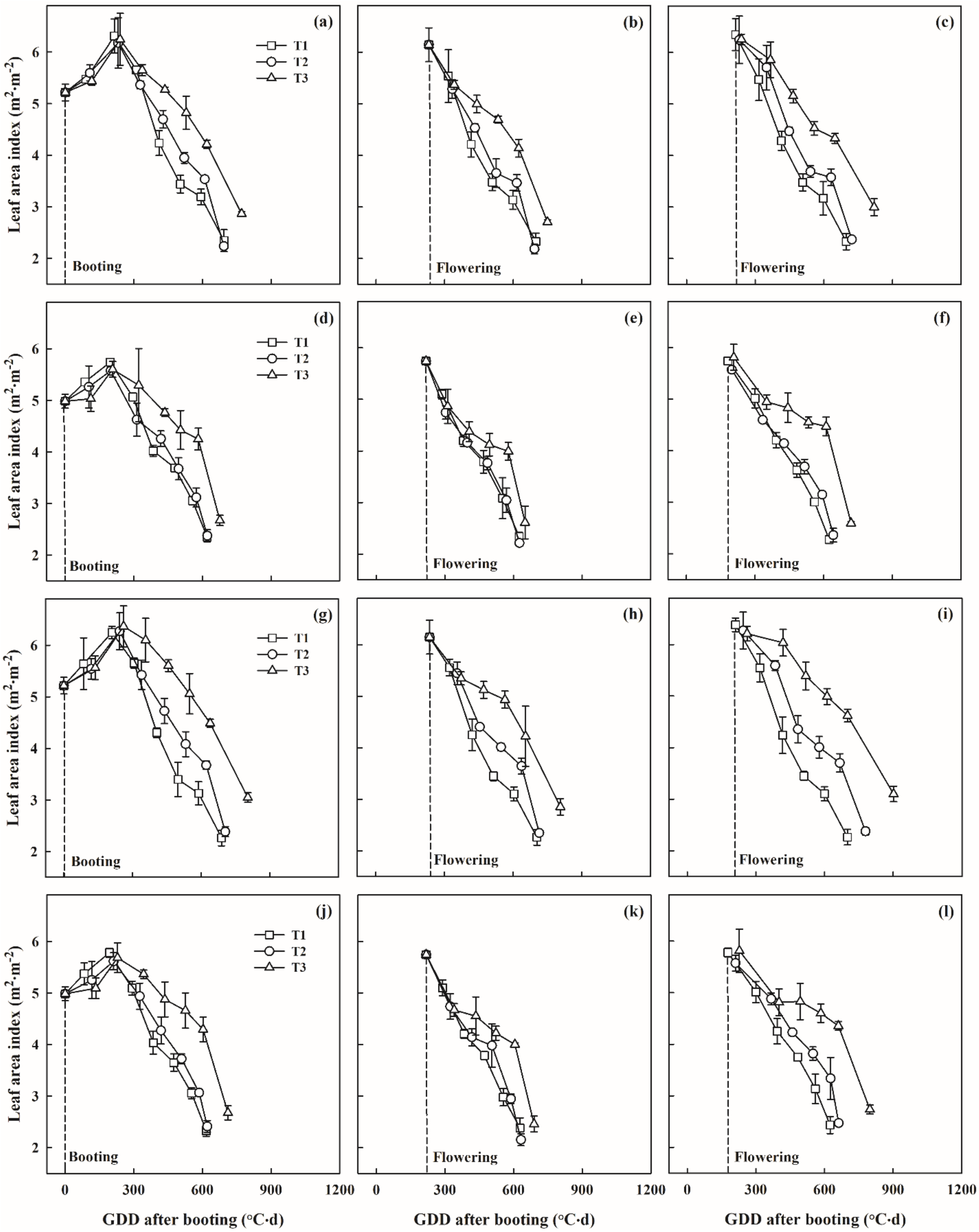

Figure S2. The measured leaf area index (LAI) under different high-temperature treatments at booting, flowering, and combined stages in 2017. (a-c): Huaidao-5 under 2 days' duration (D_2) at booting and flowering stages and under 4 days (D_{2+2}) at combined stages, respectively; (d-f): Wuyunjing-24 under 2 days' duration (D_2) at booting and flowering stages and under 4 days (D_{2+2}) at combined stages, respectively; (g-i): Huaidao-5 under 4 days' duration (D_4) at booting and flowering stages and under 8 days (D_{4+4}) at combined stages, respectively; (j-l): Wuyunjing-24 under 4 days' duration (D_4) at booting and flowering stages and under 8 days (D_{4+4}) at combined stages. Vertical bars represent standard deviation of mean.

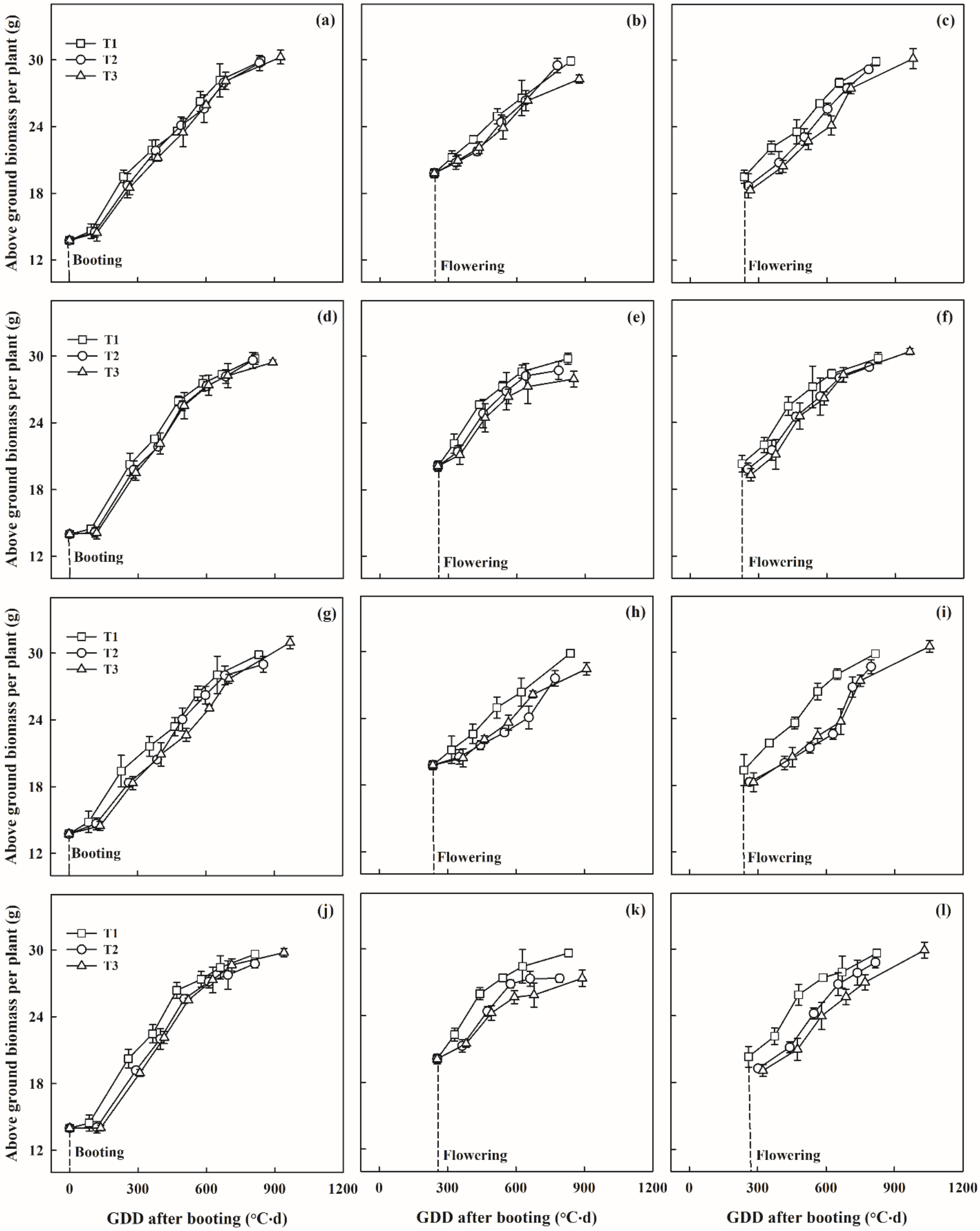

Figure S3. The measured aboveground biomass per plant (g) under different high-temperature treatments at booting, flowering, and combined stages in 2016. (a-c): Huaidao-5 under 2 days' duration (D_2) at booting and flowering stages and under 4 days (D_{2+2}) at combined stages, respectively; (d-f): Wuyunjing-24 under 2 days' duration (D_2) at booting and flowering stages and under 4 days (D_{2+2}) at combined stages, respectively; (g-i): Huaidao-5 under 4 days' duration (D_4) at booting and flowering stages and under 8 days (D_{4+4}) at combined stages, respectively; (j-l): Wuyunjing-24 under 4 days' duration (D_4) at booting and flowering stages and under 8 days (D_{4+4}) at combined stages. Vertical bars represent standard deviation of mean.

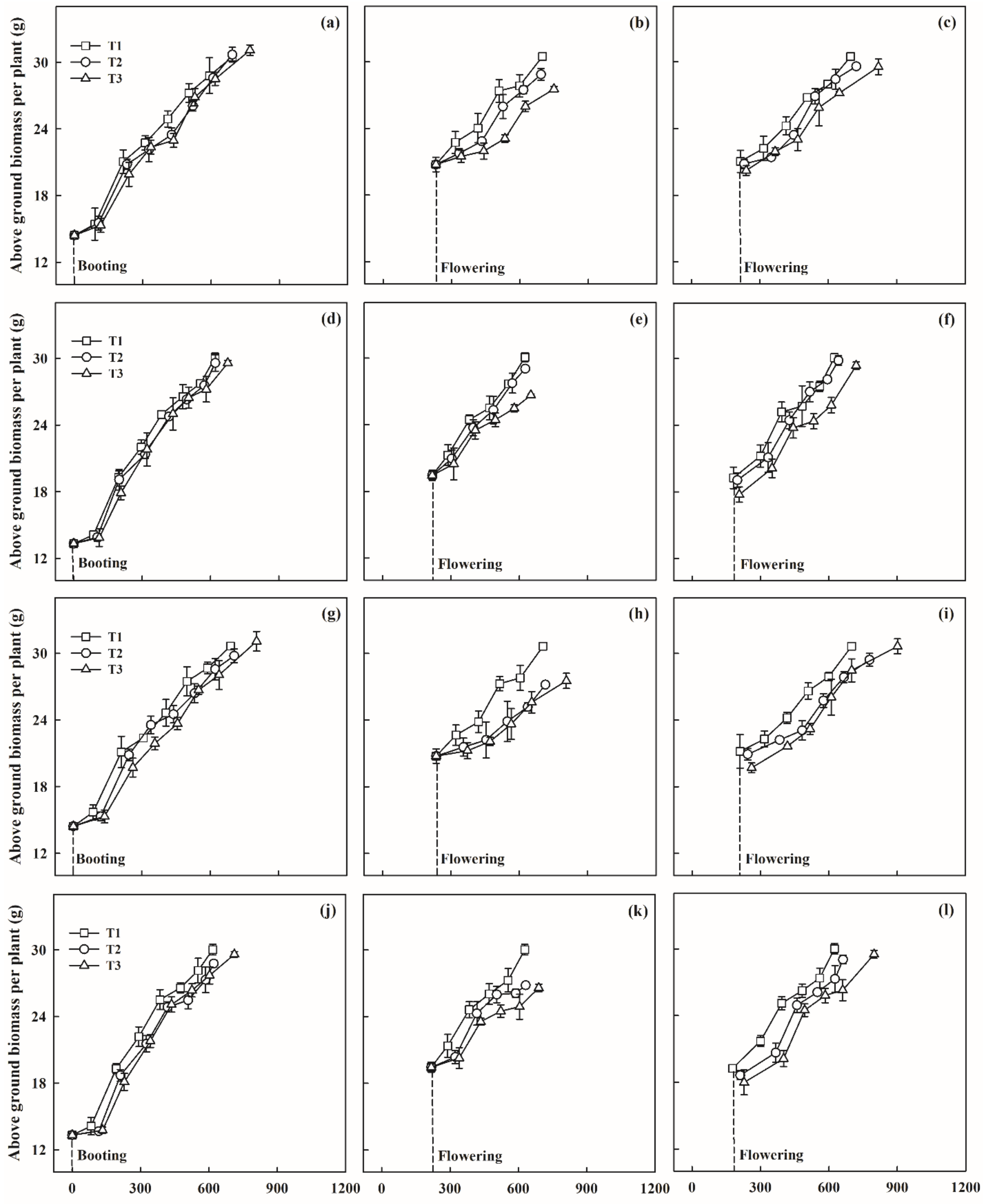

Figure S4. The measured aboveground biomass per plant (g) under different high-temperature treatments at booting, flowering, and combined stages in 2017. (a-c): Huaidao-5 under 2 days' duration (D_2) at booting and flowering stages and under 4 days (D_{2+2}) at combined stages, respectively; (d-f): Wuyunjing-24 under 2 days' duration (D_2) at booting and flowering stages and under 4 days (D_{2+2}) at combined stages, respectively; (g-i): Huaidao-5 under 4 days' duration (D_4) at booting and flowering stages and under 8 days (D_{4+4}) at combined stages, respectively; (j-l): Wuyunjing-24 under 4 days' duration (D_4) at booting and flowering stages and under 8 days (D_{4+4}) at combined stages. Vertical bars represent standard deviation of mean.

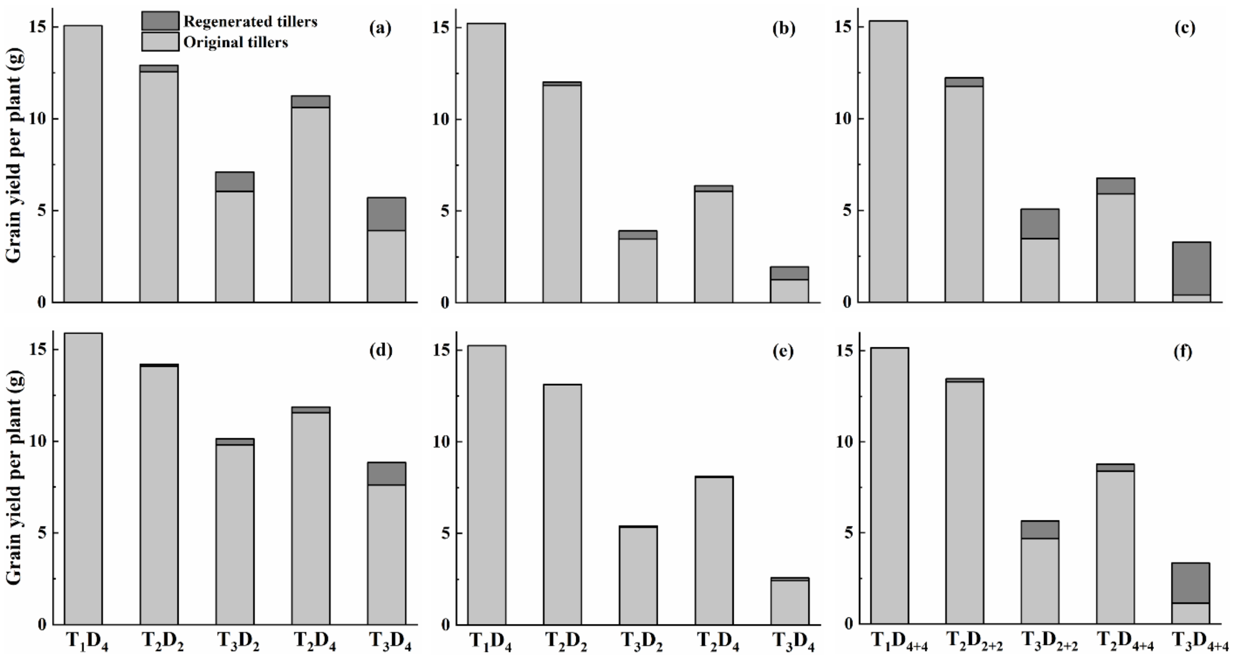

Figure S5. Grain yield per plant including original and regenerated tillers under different high-temperature treatments at booting, flowering, and combined stages during the 2016–2017 growing seasons. (a) Huaidao-5 at booting treatment; (b) Huaidao-5 at flowering treatment; (c) Huaidao-5 at combined stages treatment; (d) Wuyunjing-24 at booting treatment; (e) Wuyunjing-24 at flowering treatment; (f) Wuyunjing-24 at combined stages treatment. Different uppercase and lowercase letters indicate significant differences at the 0.05 level for original and regenerated tillers, respectively.

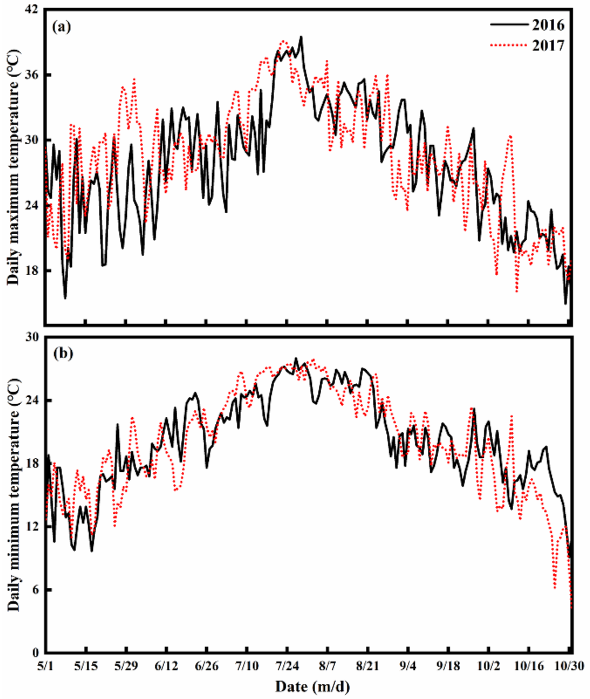

Figure S6. Daily (a) maximum and (b) minimum temperatures of the natural environment from May to October during the rice growing seasons 2016–2017.
